# Comparative Genomic Analysis Reveals the Mechanism Driving the Diversification of Plastomic Structure in Taxaceae Species

**DOI:** 10.3389/fgene.2019.01295

**Published:** 2020-01-14

**Authors:** Yue Zhang, Yang Xu, Hao Chen, Liuyang Wang, Kangquan Yin, Fang K. Du

**Affiliations:** ^1^School of Ecology and Nature Conservation, Beijing Forestry University, Beijing, China; ^2^Department of Molecular Genetics and Microbiology, School of Medicine, Duke University, Durham, NC, United States; ^3^College of Grassland Science, Beijing Forestry University, Beijing, China

**Keywords:** inversion, rearrangement, isomeric plastomes, inverted repeat, phylogenetics, yew

## Abstract

Inverted repeat (IR) regions in the plastomes from land plants induce homologous recombination, generating isomeric plastomes. While the plastomes of Taxaceae species often lose one of the IR regions, considerable isomeric plastomes were created in Taxaceae species with a hitherto unclarified mechanism. To investigate the detailed mechanism underpinning the IR-independent genesis of plastomic diversity, we sequenced four Taxaceae plastomes, including *Taxus cuspidata* Siebold & Zuccarini, *Taxus fauna* Nan Li & R. R. Mill, and two individuals of *Taxus wallichiana* Zuccarini. Then we compared these structures with those of previously reported Taxaceae plastomes. Our analysis identified four distinct plastome forms that originated from the rearrangements of two IR-flanking inverted fragments. The presence of isomeric plastomes was then verified in *T. cuspidata* individuals. Both rearrangement analyses and phylogenetic results indicated that Taxaceae were separated into two clades, one including *Taxus* and *Pseudotaxus* and another formed by *Amentotaxus* and *Torreya*. Our reconstructed scenario suggests that the minimum number of inversion events required for the transformation of the plastome of *Cephalotaxus oliveri* Masters into the diversified Taxaceae plastomes ranged from three to six. To sum up, our study reveals a distinct pattern and the mechanism driving the structural diversification of Taxaceae plastomes, which will advance our understanding of the maintenance of plastomic diversity and complexity in conifers.

## Introduction

Chloroplast (cp) is the organelle responsible for photosynthesis and providing energy for plants and photosynthetic algae (Dyall et al., 2004). Each chloroplast has its own genome (plastome) with a typical circular organization (Palmer, 1991). With the development of next-generation sequencing (NGS) and other methods for obtaining the plastomic sequences, the availability of plastome sequences has increased dramatically for land plants, offering opportunities for the comprehensive comparison and dissection of plastomic structure and variation (Du et al., 2015).

The plastomes of most land plants and algae are composed of four parts, namely, two copies of large inverted repeats (IRs) that contain four ribosomal RNA genes (*rrn16*, *rrn23*, *rrn4.5*, and *rrn5*), a large single copy (LSC) and a small single copy (SSC) region (Wang et al., 2008; Wicke et al., 2011; Davis et al., 2014). However, not all gymnosperms follow this rule. Gymnosperms are generally categorized into five groups, namely, cycads, ginkgo, gnetophytes, Pinaceae (conifers I), and cupressophytes (conifers II) (Chaw et al., 1997; Rai et al., 2008). While the plastomes of cycads, ginkgo, and gnetophytes are known to be quadripartite, recent comparative analyses of conifer plastomes have revealed that Pinaceae and cupressophyte species possess reduced IR copies. Comparative analysis have demonstrated that the Pinaceae have lost inverted repeat region B (IRB) and the cupressophytes have lost inverted repeat region A (IRA), suggesting the IR loss is homoplasious rather than synapomorphic (Wu et al., 2011; Wu and Chaw, 2014).

So far, two mechanisms have been proposed to explain the genesis of isomeric plastomes. For plants wherein the two IRs are present, the IRs can trigger homologous recombination, leading to the coexistence of two isomeric plastomes within individual plants (Palmer, 1983). However, conifers wherein only one IR is present have evolved short IRs that are associated with inversions in their plastomes (Wu and Chaw, 2016). In Pinaceae, a 40- to 50-kb inversion that can be used to distinguish *Pseudotsuga* species from that of *Pinus* has been found (Tsai and Strauss, 1989) and a 42-kb inversion was detected in *Abies* and *Tsuga* species (Tsumura et al., 2000). In cupressophytes, recombination of plastomes always occurs with a 34- to 36-kb inversion containing *trnQ*-*UUG*, as evidenced in *Cephalotaxus* (Yi et al., 2013) and *Juniperus* species (Guo et al., 2014). Although inversion has been shown to contribute to the genesis of isomeric plastomes in plants, the underlying mechanism remains not well studied (Tsumura et al., 2000; Hirao et al., 2008; Wu et al., 2011; Hsu et al., 2014; Wu and Chaw, 2014). Therefore, it is of scientific interest to elucidate the mechanism driving the diversification of plastomic organization in plants with reduced IR copies.

Taxaceae, the smallest family of conifers, belongs to the conifer II group. It consists of four genera, namely, *Amentotaxus*, *Pseudotaxus*, *Taxus*, and *Torreya* (Fu et al., 1999; Cheng et al., 2000). Some Taxaceae species have been commercially exploited for the extraction of the anticancer chemotherapeutic drug Taxol since 1990s (e.g., Poudel et al., 2013). Up to now, most of the studies concerning Taxaceae plants focused on the improvement of Taxol production (Wang et al., 2001; Li et al., 2017). For plastomic studies, genetic analyses using plastome fragments have been conducted to reconstruct the phylogeny of the Taxaceae family (e.g., Ran et al., 2010), to develop barcodes to differentiate different species (Gao et al., 2017; Liu et al., 2018), and to explore the demographic history of certain species or species complexes (Ge et al., 2015; Su et al., 2018). Recently, several groups released a number of Taxaceae plastomes (Li et al., 2016; Tao et al., 2016; Jia and Liu, 2017), and both intraspecific and interspecific isomeric arrangements have been reported (Hsu et al., 2014; Fu et al., 2019). However, the relative location and genomic organization of the inverted fragments in Taxaceae plastomes have not been comprehensively studied yet.

In this study, we firstly sequenced and analyzed four newly obtained *Taxus* plastomes from *Taxus cuspidata* Siebold & Zuccarini, *Taxus fauna* Nan Li & R. R. Mill, and two individuals of *Taxus wallichiana* Zuccarini. Next, we compared them with 11 other published Taxaceae plastomes to explore the evolutionary pattern underlying the structural diversification of Taxaceae plastomes. The information provided here will significantly advance our understanding of the functional significance of inversion and the nature of Taxaceae plastomes.

## Materials and Methods

### Sampling, DNA Extraction, and Processing

Fresh leaves were sampled from four individuals of three *Taxus* species including *T. cuspidata*, *T. fauna*, *T. wallichiana* XI3, and *T. wallichiana* XU10 (**Supplementary Table S1**). The vouchers of these species were deposited in Beijing Forestry University, Beijing, China. The chloroplast DNA was extracted and processed following the protocol developed by our group (Du et al., 2015). Next, 5 µg of the purified rolling circle amplification (RCA) product of chloroplast DNA from individual plants was used for library preparation.

### Sequencing, Assembly, and Annotation

The paired-end sequencing was performed on an Illumina-HiSeq 2000 system (Illumina, USA) by OE Biotech Co., China. For each species, more than 10 Gb of clean data were generated. We assembled the plastomes using NOVOPlasty 2.6.3.pl (Dierckxsens et al., 2017) and the plastome of *Taxus wallichiana* var. *chinensis* (Pilger) Florin (KX431996) was used as a reference (Jia and Liu, 2017). The gaps were bridged using PCR with designed primers (**Supplementary Table S2**). We annotated the sequences with CpGAVAS (Liu et al., 2012; http://47.96.249.172:16014/analyzer/home) and verified all transfer RNA (tRNA) genes using tRNAscan-SE search server (Lowe and Eddy, 1997; http://lowelab.ucsc.edu/tRNAscan-SE/). The plastome sequences were deposited in GenBank under the following accession numbers: MF095888 for the plastome of *T. cuspidata*, MF278259 for the plastome of *T. fauna*, MF850258 for the plastome of *T. wallichiana* XI3, and MG011728 for the plastome of *T. wallichiana* XU10 (**Table 1**). The circular plastome maps of the four *Taxus* species were drawn using OGDRAW (Lohse et al., 2013; https://chlorobox.mpimp-golm.mpg.de/OGDraw.html).

**Table 1:** Characteristics of 15 Taxaceae plastomes.

Genus	Species	Plastome size(bp)	Genes (protein, tRNA, rRNA)	GC content (%)	Genbank number	References
*Amentotaxus*	*A. argotaenia*	136,657	118 (83, 31, 4)	35.85	KR780582.1	Li et al. (2016)
	*A. formosana*	136,430	120 (83, 33, 4)	35.83	AP014574.1	Hsu et al. (2014)
*Pseudotaxus*	*P. chienii*	126,925	113 (82, 28, 3)	35.15	MH023407.1	Wang et al. (2019)
*Taxus*	*T. baccata*	128,653	114 (81, 29, 4)	34.59	NC_035066.1	Unpublished
	*T. cuspidata*	128,098	114 (82, 28, 4)	34.67	MF095888	This study
	*T. fuana*	128,001	114 (82, 28, 4)	34.68	MF278259	This study
	*T. mairei*	129,513	113 (82, 27, 4)	34.63	KX123824	Zhang et al. (2014)
	*T. mairei* NN014	127,665	110 (78, 28, 4)	34.72	JN867586	Hsu et al. (2014)
	*T. mairei* SNJ046	127,861	110 (77, 29, 4)	34.74	JN867591	Hsu et al. (2014)
	*T. mairei* WC052	127,717	109 (78, 27, 4)	34.76	JN867590	Hsu et al. (2014)
	*T. wallichiana* XI3	128,618	114 (82, 28, 4)	34.61	MF850258	This study
	*T. wallichiana* XU10	129,022	114 (82, 28, 4)	34.59	MG011728	This study
	*T. wallichiana* var*. chinensis*	127,743	113 (82, 27, 4)	34.71	KX431996	Jia and Liu (2017)
*Torreya*	*T. fargesii*	137,075	118 (83, 31, 4)	35.47	KT027377.1	Tao et al. (2016)
	*T. grandis*	136,949	116 (81, 31, 4)	35.44	KY369757.1	Miu et al. (2018)

### Comparison of Plastomic Structure

Fifteen Taxaceae plastomes (**Table 1**) were used to conduct comparative genomic analyses using *Cephalotaxus oliveri* Masters (KC136217) as reference (Ran et al., 2010; Ran et al., 2018). Sequences were downloaded from the National Center for Biotechnology Information (NCBI) (https://www.ncbi.nlm.nih.gov/). Dot-plot analysis between the plastomes of Taxaceae species and that of *C. oliveri* was conducted using the Blast program (https://blast.ncbi.nlm.nih.gov). In order to better understand the structure of Taxaceae plastomes, locally co-linear blocks (LCBs) among the 16 plastomes were identified using Mauve v2.4.0 (Darling et al., 2010). For this analysis, the start point of each genome was manually set to the start codon of *psbA*. The REPuter software (https://bibiserv.cebitec.uni-bielefeld.de/reputer/) was used to locate inverted repeats which flanked the inverted fragment with a minimum repeat size of 8 bp and sequence identity greater than 80% (Kurtz et al., 2001). The minimum number of inversion events required for the gene order transformations was estimated by GRIMM (Tesler, 2002; http://grimm.ucsd.edu/cgi-bin/grimm.cgi).

### Phylogenetic Analyses

To determine the evolutionary relationship of Taxaceae, we performed phylogenetic analyses using 73 protein-coding genes shared by all plastomes. After alignment by MAFFT v7 (Katoh and Standley, 2013), the third codon sites were deleted by DAMBE (Xia and Xie, 2001). We found model “GTR+I+G” is the fittest model for phylogenetic construction by jModelTest (Posada, 2008). Finally, Bayesian inference (BI) and maximum parsimony (MP) phylogenomic tree of 16 plastome sequences based on the protein-coding genes without the third codon were constructed. BI phylogenetic analyses were performed in MrBayes v3.2.3 (Ronquist and Huelsenbeck, 2003). The Markov chain Monte Carlo (MCMC) algorithm was run for 1,000,000 generations with trees sampled every 500 generations. MP phylogenetic analyses were performed in PAUP v4 (Swofford, 2003) using 1,000 bootstrap replicates.

### Detection of Isomeric Plastomes

We investigated the presence of isomeric plastomes in three *Taxus* species (**Supplementary Table S3**). Voucher specimens of these samples were deposited in Beijing Forestry University, Beijing, China. Primer pairs listed in **Supplementary Table S4** were used to amplify DNA fragments specific to the four isomeric plastomes from Taxaceae species. Each of a 25-µl PCR reaction mixture contained 1.5 µl of total genomic DNA as the template, 0.75 µl of each of the primers (10 µmol/L), 12.5 µl of 2× PCR buffer for KOD FX, 5 µl of 2 mM dNTPs, 0.5 µl of KOD FX, and 4 µl of double-distilled water. The PCR conditions were 94°C for 2 min, followed by 35 cycles of 98°C for 10 s, 56°C for 30 s, and 68°C for 1 min.

## Results

### Characteristics of the Four Newly Obtained Plastomes

**Figure 1:**
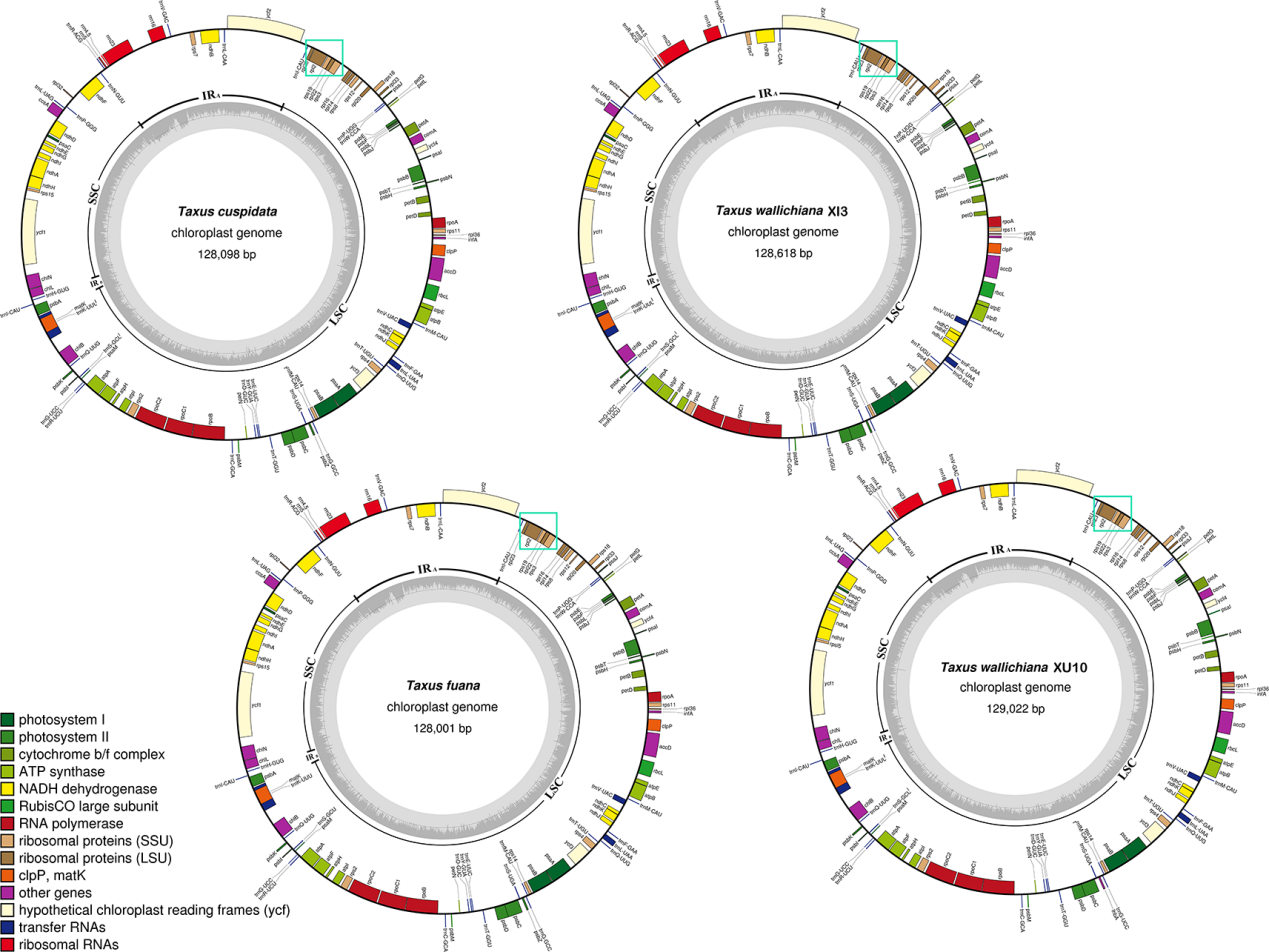
Circular map of the four newly sequenced Taxaceae plastomes. The genes that are transcribed clockwise are depicted on the *outside of the circle*, while those transcribed counterclockwise are depicted *inside*. The genes belonging to different functional groups are shown in *different colors*. *Dashed area in the inner circle* indicates the GC content of the plastomes. The *cyan solid boxes* stand for the *rpl23-rps3* clusters that are always located downstream of the IRB region.

Using *T. wallichiana* var. *chinensis* as reference (Jia and Liu, 2017), we assembled the four newly sequenced *Taxus* plastomes with NOVOPlasty 2.6.3.pl (Dierckxsens et al., 2017) and obtained circular sequences of size ranging from 128,001 to 129,022 bp (**Table 1** and **Figure 1**). Annotation by CpGAVAS and tRNAscan-SE search server suggests that each plastome contained 82 protein-coding genes, 28 tRNA genes, and four ribosomal RNA (rRNA) genes. Among these genes, only *trnI*-*CAU* and *trnQ*-*UUG* had two copies. When compared with the other six *Taxus* plastomes, namely, *Taxus baccata*, *Taxus mairei*, *T. mairei* SNJ046, *T. mairei* WC052, *T. mairei* NN014, and *T. wallichiana* var. *chinensis*, these 10 plastomes were comparable regarding plastome size, gene number, and guanine–cytosine (GC) content (34.59–34.76%; **Table 1**). Overall, the contents of protein-coding genes were relatively consistent among Taxaceae species. The plastomes of *Amentotaxus* and *Torreya* species were larger than 130 kb and harbored more genes (**Table 1**). However, the plastome of *Pseudotaxus chienii* was smaller and contained only three rRNA genes (*rrn4.5*, *rrn5*, and *rrn23*), suggesting that it might have evolved toward reduced size (Wu and Chaw, 2014; Wu and Chaw, 2016).

### Diversification in the Structure of Taxaceae Plastomes

In this study, we found that the *rpl23-rps3* cluster (cyan solid boxes in **Figure 1**) was adjacent to the unique rRNA operon (*rrn16*, *rrn23*, *rrn4.5*, and *rrn5*). Through a careful check of the conserved gene order as suggested by Wu et al. (2011), we further determined that the lost IR copy was likely to be IRA, rather than IRB.

**Figure 2:**
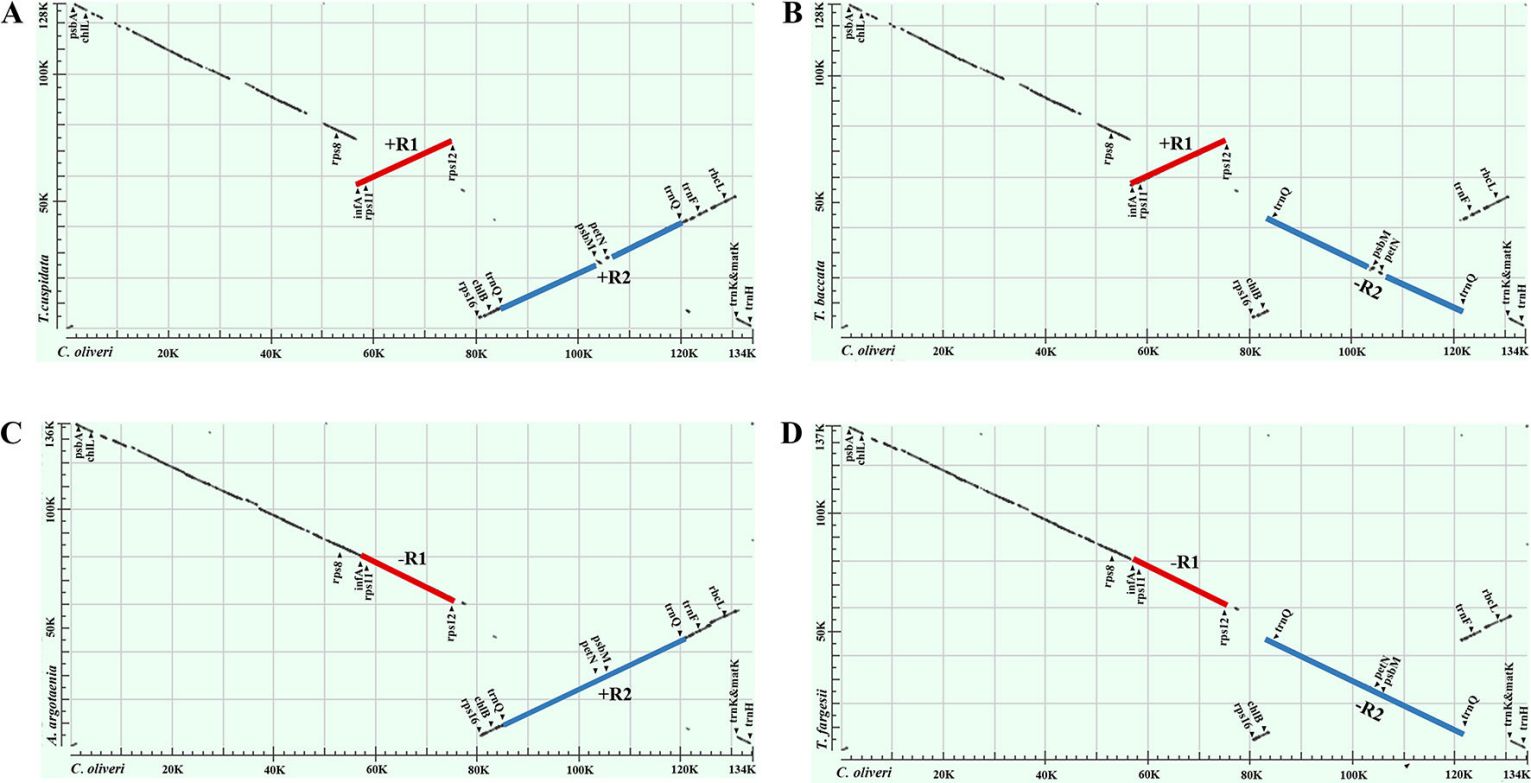
Dot-plot analyses of the four Taxaceae plastomes. The *Cephalotaxus oliveri* plastome (*horizontal axes*) was used as the reference. A positive slope denotes that the plastomic sequences of the two species can be aligned in the same orientation, whereas a negative one indicates that the two sequences are in the opposite orientations. Labeling of the genes is based on their corresponding positions in the *C. oliveri* plastome. The *red line* represents the region between *infA* and *rps12* and the *blue line* represents the region between *trnQ*-IRs. These two regions were termed R1 and R2, respectively. The form **(A)** plastomes were characterized by +R1 and +R2, the form **(B)** plastomes were characterized by +R1 and −R2, the form **(C)** plastomes were characterized by −R1 and +R2 and the form **(D)** plastomes were characterized by −R1 and −R2.

**Figure 3:**
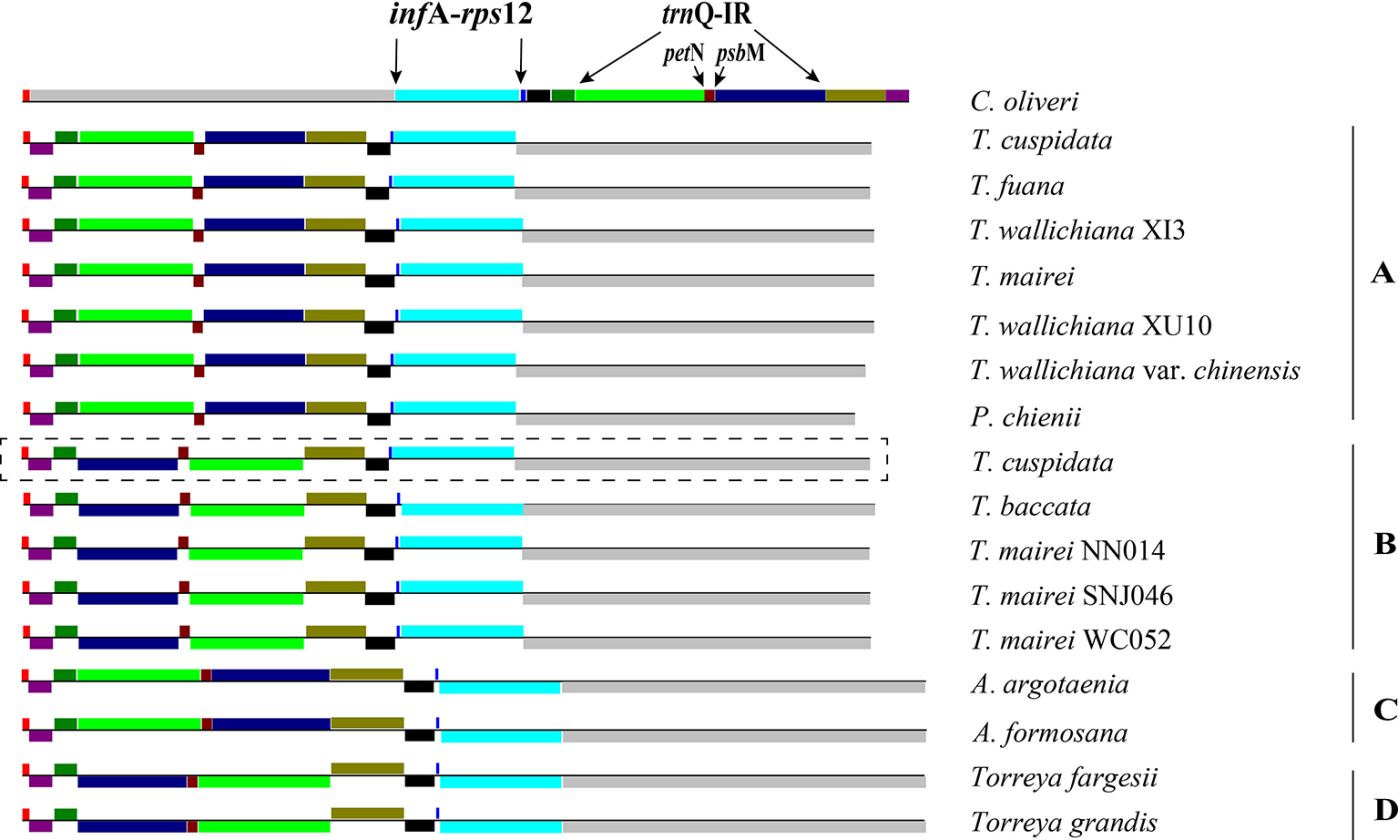
Structures of Taxaceae plastomes. The 11 locally collinear blocks (LCBs) are depicted with *different colors on the left side*. LCBs *below the horizontal line* represent opposite orientations when compared to that of *Cephalotaxus oliveri*. **(A**–**D)** after species indicates the categorization of its plastomic structure. Plastome form of *Taxus cuspidata* that showed in the *rectangle with dashed lines* represents the structure discovered by PCR. Plastome form of *T. cuspidata* that showed *without the rectangle* is based on the plastome sequence assembled from next-generation sequencing data.

Our dot-plot analyses indicated that there were two inverted fragments. Fragment of *infA*-*rps12* was approximately 18 kb in length and hereafter termed as R1. Fragment of *trnQ*-IR was approximately 34 kb in length and resulted from the full duplication of *trnQ*-*UUG* gene, so it was hereafter termed as R2. A relocated fragment of *petN*-*psbM* in R2 separated R2 into two parts, namely, *psbK* to *trnC*-*GCA* (~18 kb) and *trnD*-*GUC* to *trnT*-*UGU* (~16 kb) (**Figures 2** and **3** and **Supplementary Figure 1**).

**Figure 4:**
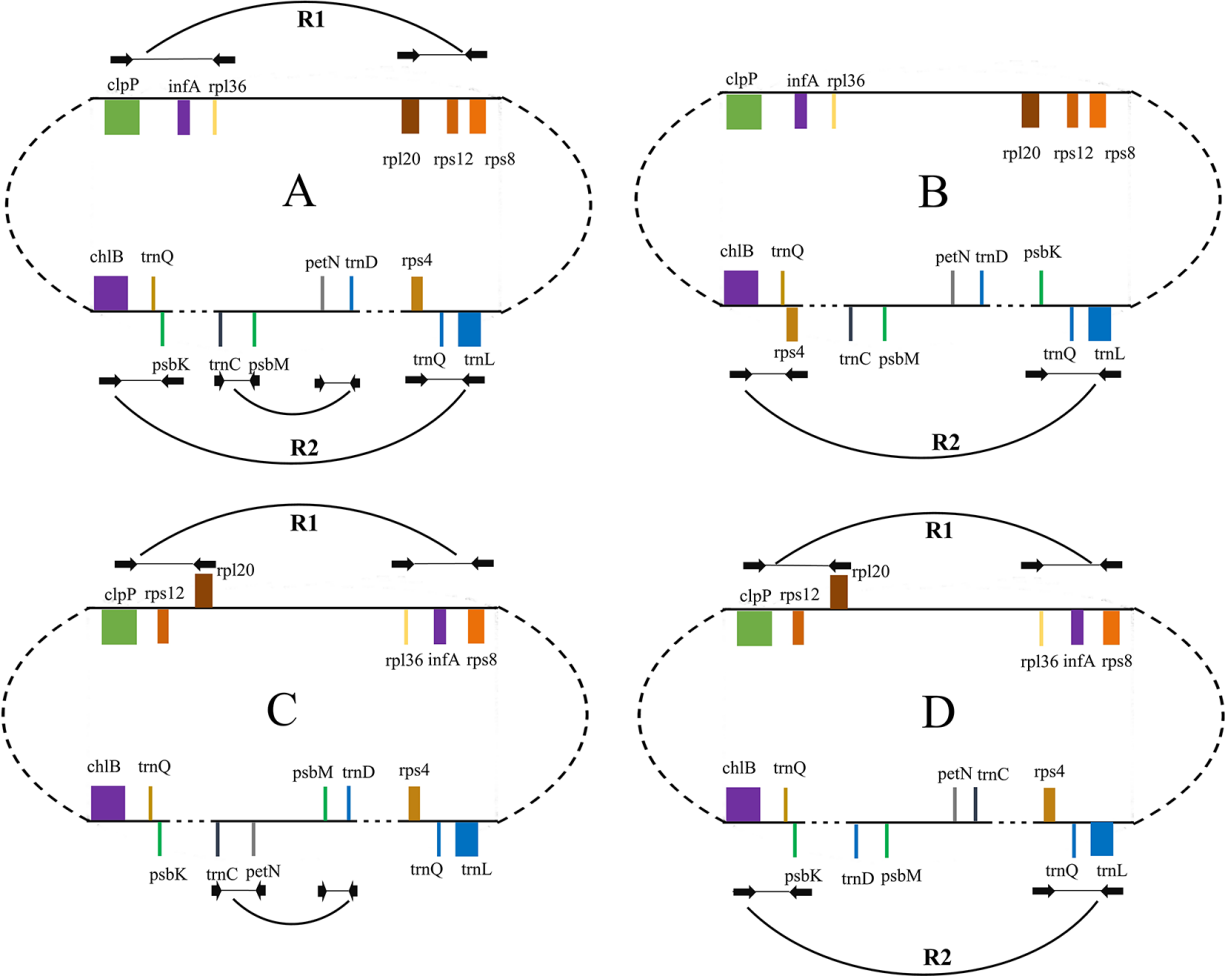
Schematic representation of different forms of isomeric plastomes in *Taxus cuspidata* individuals. Form **(A)** is the map of the plastomes obtained in our genome assembly shown in **Figure 1**. Form **(B)** differs from form **(A)** by an inversion of *trnQ*-IR fragment. Forms **(A)** and **(C)** differ by the inversion of *infA-rps12* and *petN*-*psbM* fragments. Form **(D)** differs from form **(A)** by the inversion of *infA-rps12* and *trnQ*-IR fragments. The paired PCR primers designed for the detection of isomeric plastomes are shown as *black arrows*. Ends of these inversion fragments are connected with *breaking arcs*.

We categorized the Taxaceae plastomes into four forms, namely, A, B, C, and D, based on the relative location and orientation of R1 and R2 (**Figure 2** and **Supplementary Figure 1**). Plastome assembly form A is considered as the reference hereafter. Form B plastomes carried an inverted R2, resulting in increased adjacencies between *chlB* and *rps4*, *psbK* and *trnL* and reduced adjacencies between *chlB* and *psbK*, *rps4* and *trnL* (**Figure 4**). Form C plastomes differed from those of form A in the arrangement of R1 and *petN*-*psbM* and exhibited increased adjacencies between *clpP* and *rps12*, *infA* and *rps8*, *trnD* and *psbM*, and *petN* and *trnC* and reduced adjacencies between *clpP* and *infA*, *rps12* and *rps8*, *trnC* and *psbM*, and *petN* and *trnD* (**Figure 4**). Form D plastomes had R1 and R2 arrangements distinct from those of the other three forms.

To better understand the plastome organization of Taxaceae species, we compared the plastomes of 15 Taxaceae species or varieties with that of *C. oliveri* and determined 11 LCBs (**Figure 3**). We found the form A plastomes were characterized by +R1 and +R2 (+ denotes the forward strand and − denotes the reverse strand); for example, in *Pseudotaxus chienii* W. C. Cheng and six other *Taxus* species or varieties, namely, *T. cuspidata*, *T. fauna*, *T. mairei*, *T. wallichiana* var. *chinensis*, *T. wallichiana* XI3, and *T. wallichiana* XU10. The form B plastomes, characterized by +R1 and −R2, were found in four *Taxus* species, namely, *T. baccata*, *T. mairei* NN014, *T. mairei* SNJ046, and *T. mairei* WC052. The form C plastomes, characterized by −R1 and +R2, were found in *Amentotaxus argotaenia* Pilger and *Amentotaxus formosana* H. L. Li. The form D plastomes were characterized by −R1 and −R2 and were found in *Torreya fargesii* Franchet and *Torreya grandis* Fortune. Among the 15 Taxaceae plastomes, we observed that the organization of *T. mairei* plastome was of form A and the plastomes from three Taiwan *T. mairei* individuals (NN014, SNJ046, and WC052) were of form B, indicating that plastomes of these two forms are present in *T. mairei* (**Figure 3**).

### Experimental Validation of the Four Arrangement Forms

We next performed PCR to verify the presence of these arrangement forms in plants of Taxaceae species (**Figure 4**). We found that 20 *T. cuspidata* individuals from 20 natural populations harbored two isomeric plastomes, namely, forms A and B, but not forms C and D (**Figure 3**, **Supplementary Figure 2**, and **Supplementary Table S5**). However, we did not find isomeric plastomes in *T. fauna* and *T. wallichiana*.

### Evolutionary Path of Plastome IR Formation in Taxaceae

**Figure 5:**
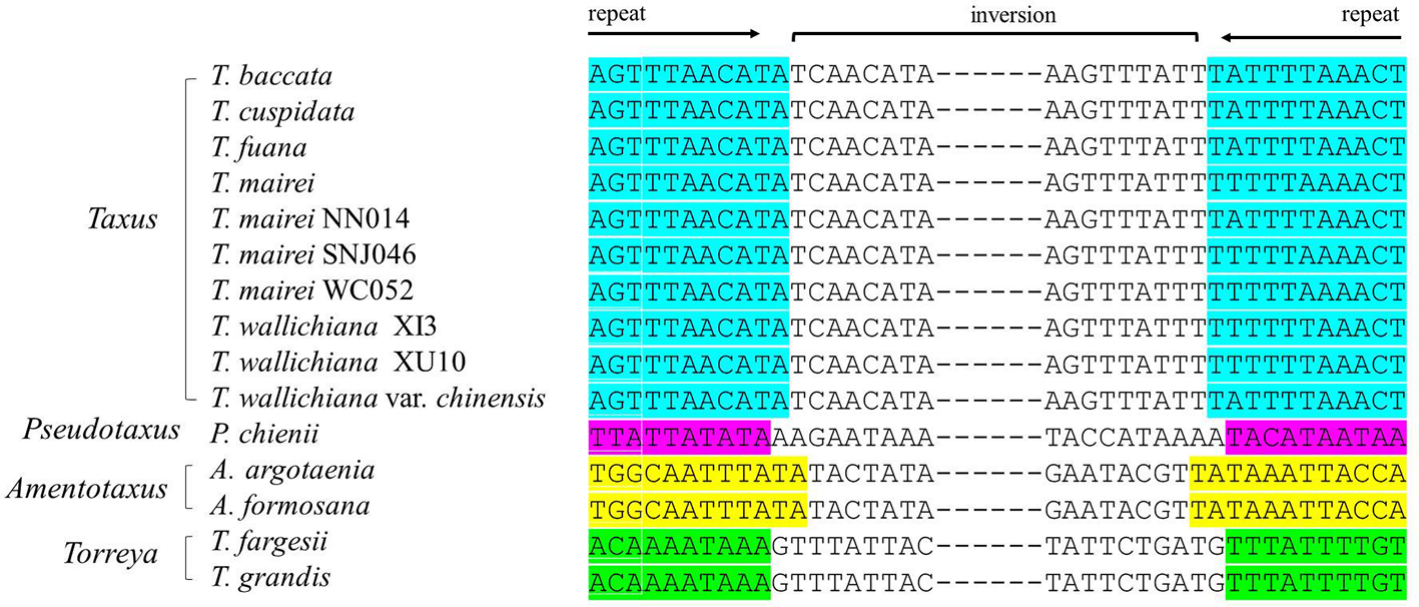
Pairs of short inverted repeats at the edges of the *infA*-*rps12* fragment in Taxaceae plastomes. Repeats are represented using *different colors*.

We detected several pairs of 9- to 12-bp inverted short repeats that specifically flanked the region of R1 in Taxaceae species, and these repeats were similar to those in species within the same genus (**Figure 5**). However, we failed to detect any inverted short repeats larger than 9 bp within the *petN*-*psbM* fragment. The inverted fragment R2 was flanked by the *trnQ*-IR that resulted from the full duplication of the *trnQ*-*UUG* gene of which the length ranged from 114 to 560 bp in the 15 Taxaceae plastomes (**Table 2**).

**Table 2:** Inverted repeats of *trnQ*-IR in 15 Taxaceae plastomes.

Genus	Species	*trnQ*-IR length		Repeat start 1	Repeat start 2	Location of repeat 1	Location of repeat 2
*Amentotaxus*	*A. argotaenia*		527	7,055	46,099	*chlB*	*trnT-UGU*/*trnQ-UUG*
	*A. formosana*		560	7,052	46,073	*chlB*	*trnT*/*trnQ*
*Pseudotaxus*	*P. chienii*		552	76,136	113,001	*chlB*	*trnT-UGU*/*trnQ-UUG*
*Taxus*	*T. baccata*		114	7,015	42,868	*psbB*	*rrn5*/*trnR-ACG*
	*T. cuspidata*		238	78,235	113,927	*chlB*/*trnQ-UUG*	*trnT-UGU*/*trnQ-UUG*
	*T. fauna*		230	77,951	113,653	*chlB*/*trnQ-UUG*	*trnT-UGU*/*trnQ-UUG*
	*T. mairei*		248	78,809	114,654	*chlB*/*trnQ-UUG*	*trnT-UGU*/*trnQ-UUG*
	*T. mairei* NN014		114	7,608	43,347	*chlB*/*trnQ-UUG*	*psbK*/*trnQ-UUG*
	*T. mairei* SNJ046		248	7,620	43,237	*chlB*/*trnQ-UUG*	*psbK*/*trnQ-UUG*
	*T. mairei* WC052		248	7,621	43,265	*chlB*/*trnQ-UUG*	*psbK*/*trnQ-UUG*
	*T. wallichiana* XI3		239	78,613	114,490	*chlB*/*trnQ-UUG*	*trnT-UGU*/*trnQ-UUG*
	*T. wallichiana* XU10		239	78,979	114,870	*chlB*/*trnQ-UUG*	*trnT-UGU*/*trnQ-UUG*
	*T. wallichiana* var*. chinensis*		209	77,941	113,598	*chlB*/*trnQ-UUG*	*trnT-UGU*/*trnQ-UUG*
*Torreya*	*T. fargesii*		298	7,429	46,543	*chlB*	*psbK*/*trnQ-UUG*
	*T. grandis*		298	7,437	46,614	*chlB*	*psbK*/*ndhJ*

**Figure 6:**
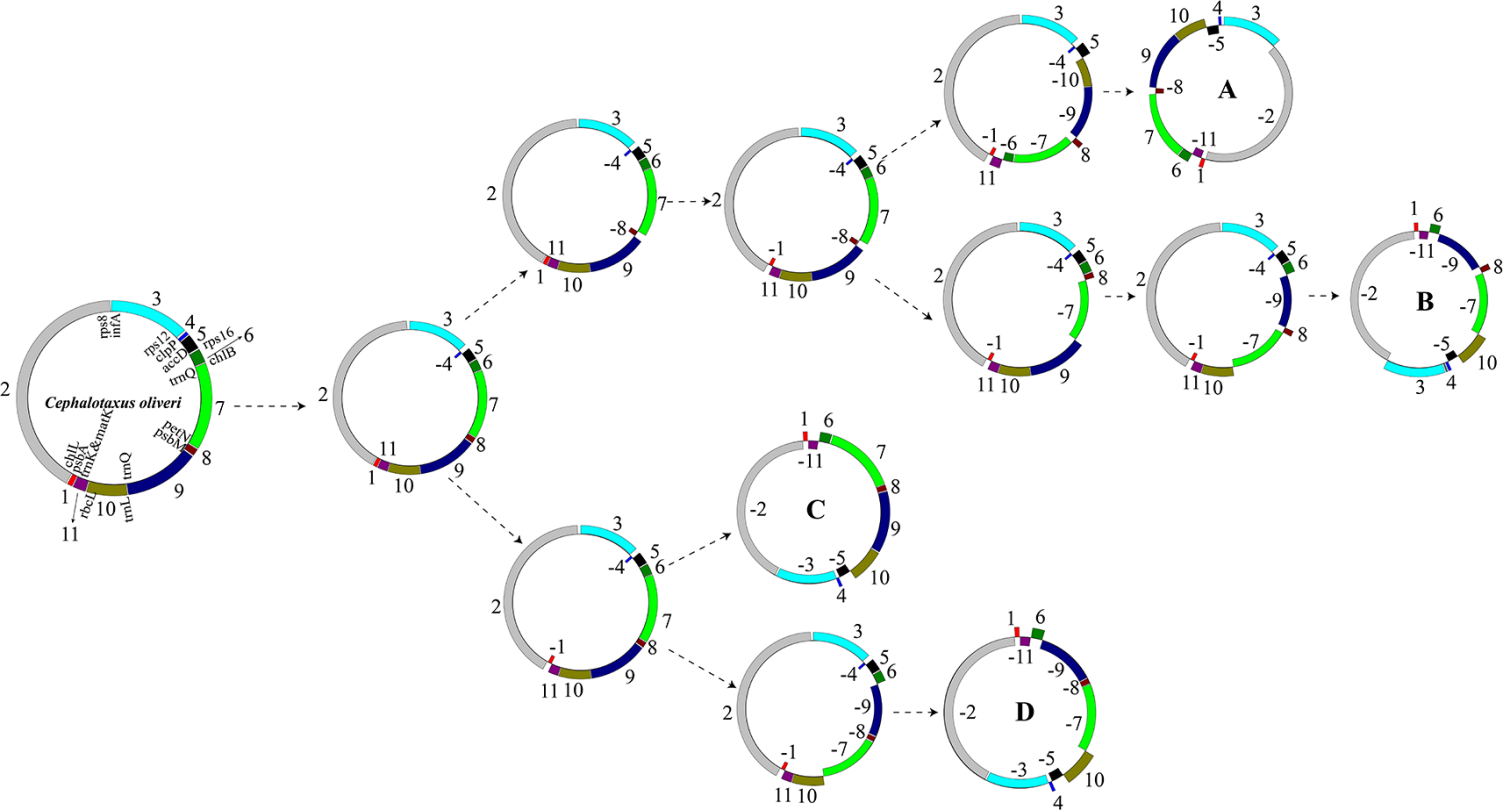
The putative transformation from the plastome of *Cephalotaxus oliveri* to the four diversified Taxaceae plastomes. Eleven locally collinear blocks (LCBs) were denoted using *different colors*. Gene orientations were shown by placing them *inside* or *outside of the circles*. The minimum number of inversion events required for the transformation from the plastome of *C. oliveri* to the Taxaceae plastomes was calculated using GRIMM.

As aforementioned, we identified 11 LCBs among the plastomes of the 15 Taxaceae individuals (**Figure 3**). The arrangements of these LCBs and that of *C. oliveri* were used to infer the most parsimonious rearrangement scenario that will provide information for the dissection of plastomic inversion history in Taxaceae species. The minimum number of inversion events required for the transformation from the *C. oliveri* plastome to the plastomes of forms A, B, C, and D was five, six, three, and four, respectively (**Figure 6**). As for the first transformation, the inversion of block 4 (*clpP*) took place, and then the inversion of block 8 (*petN*-*psbM*) occurred in one clade, generating the *Taxus* and *Pseudotaxus* plastomes. The other clade underwent the inversion of block 1 (*psbA*) and became the *Amentotaxus* and *Torreya* plastomes.

A total of 41,596 aligned sites, including 5,357 variable and 2,849 parsimony-informative sites, were used to reconstruct the phylogenetic tree. MP and BI results both suggest that Taxaceae are separated into two clades: one including *Taxus* and *Pseudotaxus* and another formed by *Amentotaxus* and *Torreya* at the basal position (**Supplementary Figure 3**). These phylogenetic relationships are consistent with our GRIMM analysis results which also recovered these two clades (the A/B and C/D plastome forms) (**Figure 6**). Additionally, species from the same genus were placed on a single clade. However, neither *T. wallichiana* nor *T. mairei* appear to be monophyletic species in our tree (**Supplementary Figure 3**).

## Discussion

Issues related to the IR region have been a research focus for several decades (Kolodner and Tewari, 1979; Zhu et al., 2016). In recent years, the rapidly increasing genomic resources of plants in the five gymnosperm groups have revealed considerable variations in plastomic structure (Raubeson and Jansen, 1992; Wu et al., 2009; Lin et al., 2012). The presence of only one IR copy in Pinaceae and Cupressophytes species was considered as a homologous character (Raubeson and Jansen, 1992). However, recent comparative analyses of conifer plastomes uncovered distinct evolutionary patterns in Pinaceae and cupressophytes plastomes, namely, the former lost IRB and the latter lost IRA (Wu et al., 2011; Hao et al., 2016). Due to the presence of isomeric plastomes, it has been difficult to determine which IR copy was lost (Yi et al., 2013). In this study, using a comparative plastome profiling, we revealed that the plastomes of all the 12 species from four different genera of Taxaceae contained an *rpl23-rps3* cluster that is located downstream of the IRB, thus demonstrating that Taxaceae plastomes lack IRA (**Figure 1**).

IRs trigger homologous recombination and the coexistence of two isomeric plastomes has been found at the infraspecific level in numerous species (Palmer, 1983). Despite the absence of IRs, conifers contain isomeric plastomes that are produced by inversions, and these inversions are mediated by specific short IRs (Wu and Chaw, 2016). In our study, we revealed four new forms of organization in Taxaceae plastomes, which have possibly been generated by the rearrangements of two large inverted fragments (R1: *infA*-*rps12* and R2: *trnQ*-IR) that are flanked by specific short repeats (**Figure 5** and **Table 2**). We experimentally verified the presence of the isomeric arrangements of R2 inversion in *T. cuspidata*. However, we failed to identify isoforms in *T. fauna* and *T. wallichiana*. Diversified plastomic structure has also been found to be ubiquitous in other conifers. Four distinct types, generated by two inverted fragments (F1: *trnR*-*UCU* to *trnE*-*UUC*; F2: *rps4* to *trnG*-*GCC*), have been uncovered in Pinaceae, and these two inverted fragments are flanked by Pinaceae-specific repeats (Tsumura et al., 2000; Wu et al., 2011). In cupressophytes, several types of inversion have been discovered. For example, a 36-kb inversion mediated by the 216- to 552-bp *trnQ*-IR has been found in the isomeric plastomes of *Cephalotaxus* (Yi et al., 2013), *Juniperus* (Guo et al., 2014), *Pseudotaxus*, and *Taxus* species (Fu et al., 2019). Besides, a 73-kb inversion in the isomeric plastomes of *Sciadopitys* species is mediated by a 370-bp IR (Hsu et al., 2016), while the isomeric plastomes of *Calocedrus* species are found to harbor a 34-kb *rpl23*-*trnL/CAA* inversion mediated by an 11-bp IR (Qu et al., 2017). Dealing with the rearrangements of two specific inverted fragments, our study provides novel insights into the structural diversification of Taxaceae plastomes.

Phylogeny of the Taxaceae genera has been controversial for many years (Lu et al., 2014; Wu and Chaw, 2016; Zhang et al., 2019). In this study, 73 shared protein-coding genes were used to determine the relationships among the four genera. We successfully obtained a high resolution of evolutionary relationships for the four genera and placed them on two clades of Taxaceae. Both BI and MP trees showed *Amentotaxus* and *Torreya* to branch just after the outgroup (**Supplementary Figure 3**). Our results are consistent with the phylogenic tree based on the *rps3* gene (Ran et al., 2010) and transcriptomic data (Ran et al., 2018). Our rearrangement analyses by GRIMM provided the most parsimonious evolutionary scenario, which is consistent with the phylogenic tree (**Figure 6**).

It should be noted that several issues remain to be addressed. For example, the identification of the diversified plastomic structures here was based on the published Taxaceae plastomic sequences. Whether these diversified structures exist in other uncharacterized Taxaceae species needs further investigation. Besides, the presence of isomeric plastomes in Taxaceae species was only verified within only a few individuals. At the population level, however, their distribution remains unknown. Further exploration, such as determining whether there is a bias in the structure of plastome at the population level, will surely provide more information regarding the evolution of plastomes.

## Conclusions

Using next-generation sequencing and *de novo* assembly, we obtained the complete plastomes of four individuals from three *Taxus* species and profiled their structures along with 11 published Taxaceae plastomes. Our analyses revealed that in Taxaceae plastomes, the rearrangements of two large fragments could lead to the genesis of the four distinct plastome forms. Further experiments verified the presence of two isomeric plastomes in *T. cuspidata* individuals. Based on these findings, both rearrangement analyses and phylogenetic results indicated that Taxaceae were separated into two clades, one including *Taxus* and *Pseudotaxus* and another formed by *Amentotaxus* and *Torreya*. These findings highlight that the evolution of plastomes may be more complicated than previously thought. The information provided here will significantly advance our understanding of the dynamic and complex evolution of plastomes in conifers.

## Data Availability Statement

The datasets generated for this study can be found in NCBI SRA BioProject IDs PRJNA554197, PRJNA554072, PRJNA554062 and PRJNA553347.

## Author Contributions

FD designed the study. YZ, YX, and HC performed the analysis. FD, YZ, KY and LW wrote the manuscript. All the authors revised the manuscript.

## Funding

This work was supported by the National Key Research and Development Plan “Research on protection and restoration of typical small populations of wild plants” (grant no. 2016YFC0503106), Fundamental Research Funds for the Central Universities (no. 2015ZCQ-LX-03), and the National Science Foundation of China (grant 41671039) to FD.

## Conflict of Interest

The authors declare that the research was conducted in the absence of any commercial or financial relationships that could be construed as a potential conflict of interest.

## Supplementary Material

Supplementary Figure S1 Comparative dot-plot analyses between the plastome of Cephalotaxus oliveri and the four Taxaceae plastomes. A positive slope denotes that the two sequences (horizontal and vertical axes) are matched in the same orientation, whereas a negative one indicates that the two sequences can be aligned but in the opposite orientations. Genes are labeled based on their corresponding positions in the C. oliveri plastomes.

Supplementary Figure S2 The PCR results of the verification of isomeric plastomes in 20 individuals of T. cusipidata. The codes in the picture stand for different combination of individuals and primer pairs can be found in **Table S5**.

Supplementary Figure S3 Phylogenetic relationships of the Taxaceae plastomes. The trees inferred from Bayesian inference (BI) and Maximum Parsimony (MP) are based on a nucleotide supermatrix of 73 shared protein-coding genes excluding the third codon positions. The BI topology is shown with BI posterior probability/MP bootstrap values given at each node.
