# Supplementary figures and images for: Comparative Genomic Analysis Reveals the Mechanism Driving the Diversification of Plastomic Structure in Taxaceae Species
Source: Front Genet. 2020 Jan 14;10:1295. doi: 10.3389/fgene.2019.01295 (PMC6971195; doi:10.3389/fgene.2019.01295)

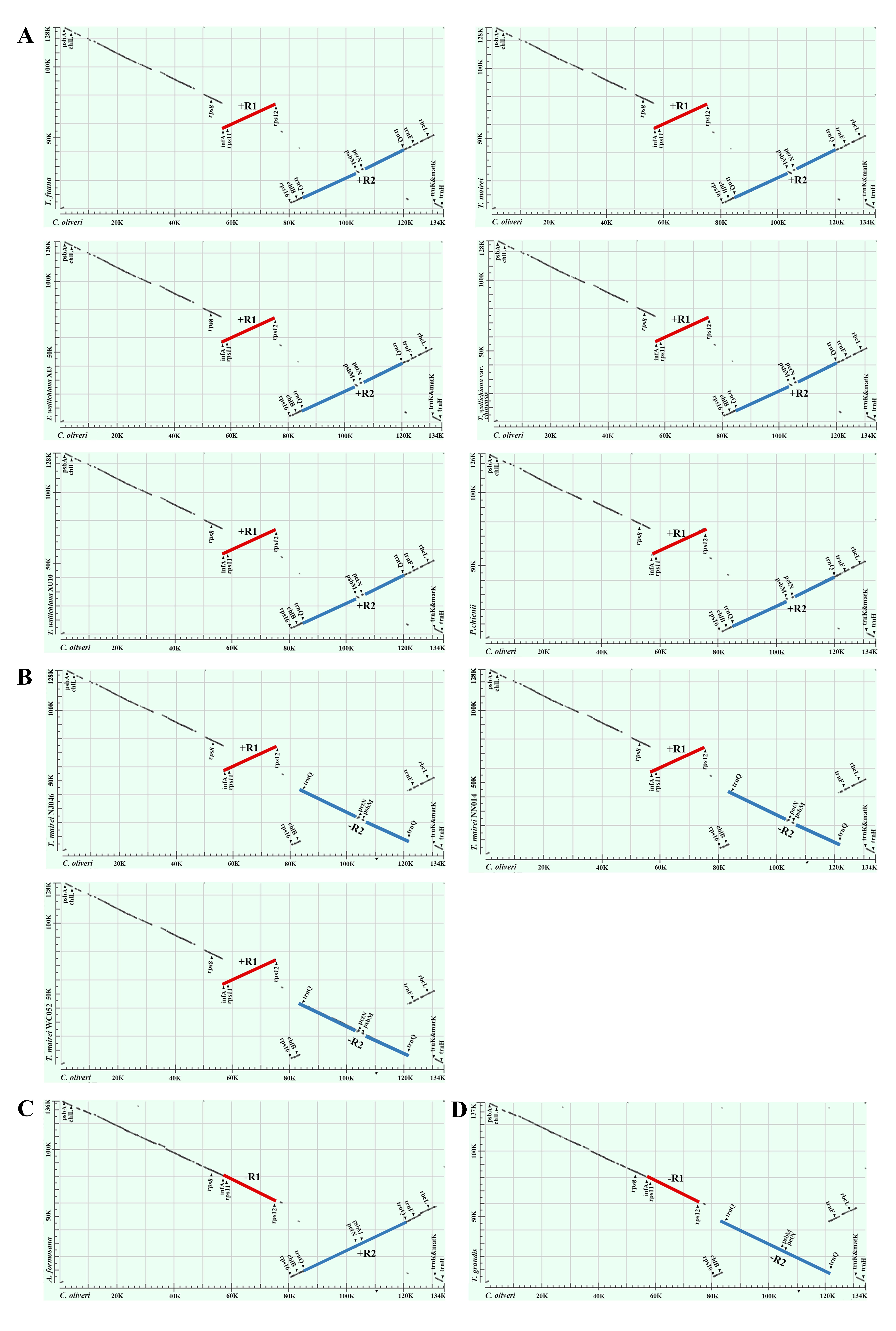

Supplement: Supplementary Figure S1 — Comparative dot-plot analyses between the plastome of Cephalotaxus oliveri and the four Taxaceae plastomes. A positive slope denotes that the two sequences (horizontal and vertical axes) are matched in the same orientation, whereas a negative one indicates that the two sequences can be aligned but in the opposite orientations. Genes are labeled based on their corresponding positions in the C. oliveri plastomes. [file Image_1.jpeg]

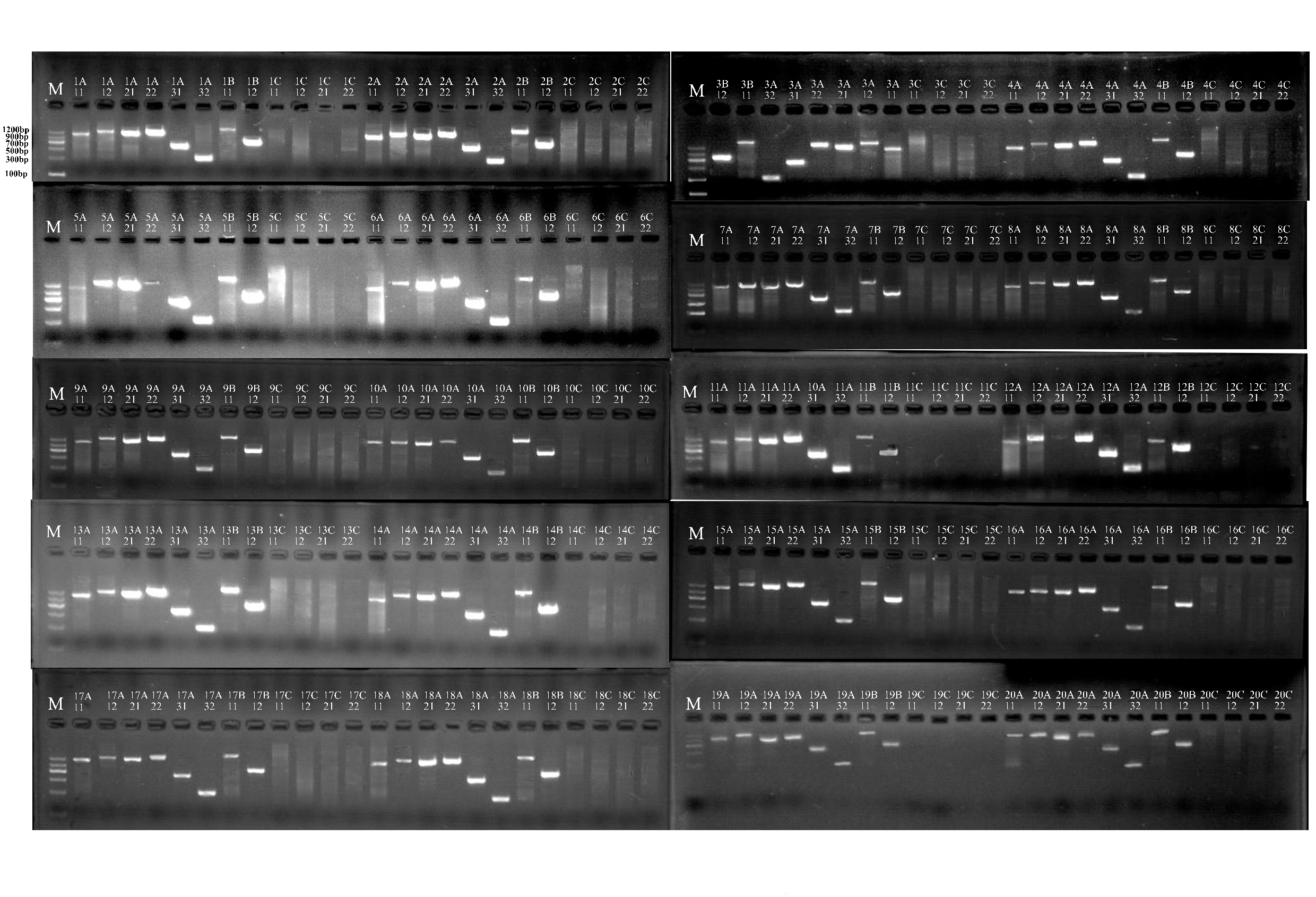

Supplement: Supplementary Figure S2 — The PCR results of the verification of isomeric plastomes in 20 individuals of T. cusipidata. The codes in the picture stand for different combination of individuals and primer pairs can be found in Table S5. [file Image_2.jpeg]

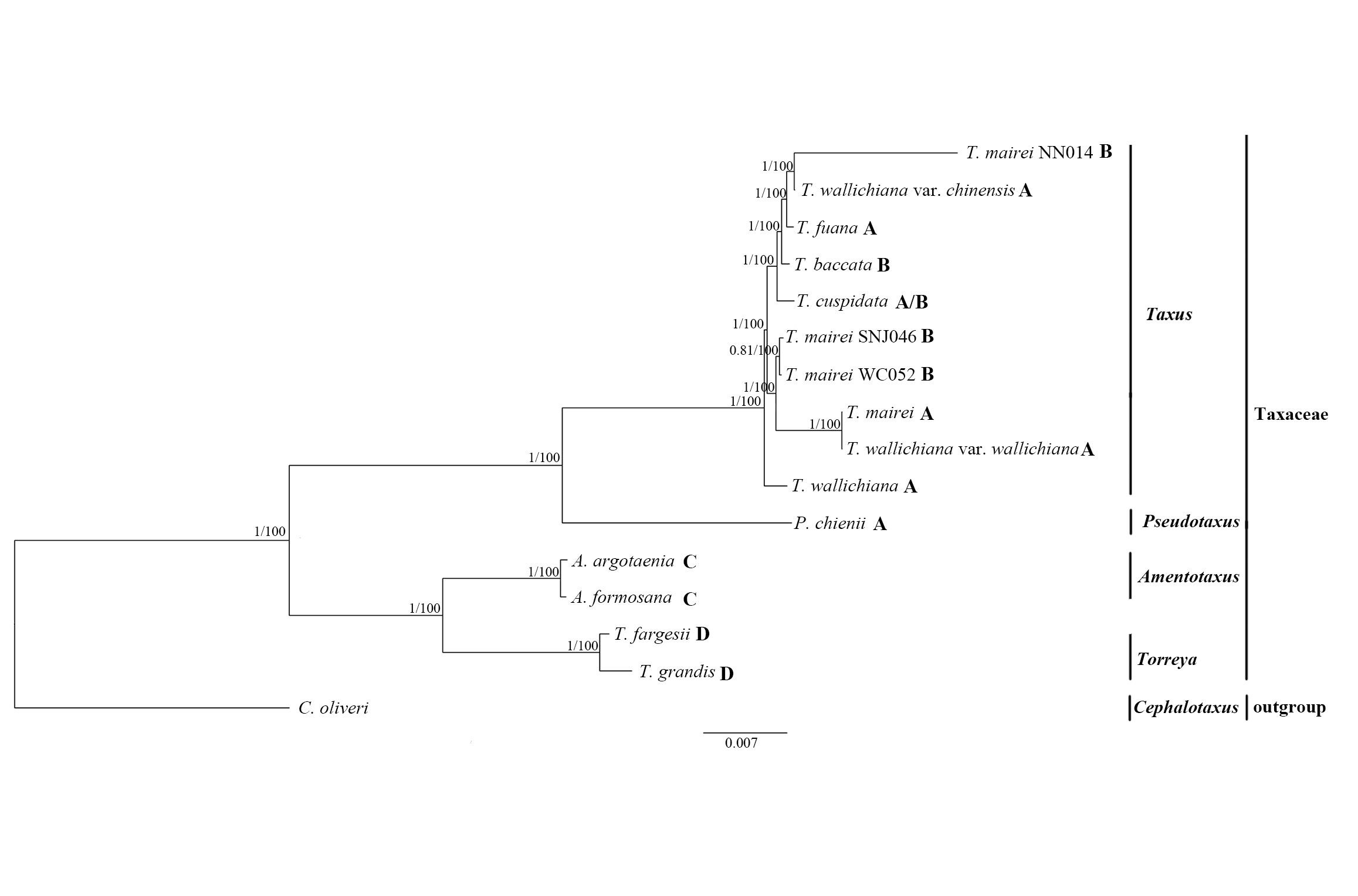

Supplement: Supplementary Figure S3 — Phylogenetic relationships of the Taxaceae plastomes. The trees inferred from Bayesian inference (BI) and Maximum Parsimony (MP) are based on a nucleotide supermatrix of 73 shared protein-coding genes excluding the third codon positions. The BI topology is shown with BI posterior probability/MP bootstrap values given at each node. [file Image_3.jpeg]
